# Oxidation of a non-phenolic lignin model compound by two *Irpex lacteus* manganese peroxidases: evidence for implication of carboxylate and radicals

**DOI:** 10.1186/s13068-017-0787-z

**Published:** 2017-04-21

**Authors:** Xing Qin, Xianhua Sun, Huoqing Huang, Yingguo Bai, Yuan Wang, Huiying Luo, Bin Yao, Xiaoyu Zhang, Xiaoyun Su

**Affiliations:** 1 College of Life Science and Technology, Huazhong University of Science and Technology, Wuhan, 430074 People’s Republic of China; 2 Key Laboratory for Feed Biotechnology of the Ministry of Agriculture, Feed Research Institute, Chinese Academy of Agricultural Sciences, No. 12 South Zhongguancun Street, Beijing, 100081 People’s Republic of China

**Keywords:** *Irpex lacteus*, Manganese peroxidase, Non-phenolic lignin, Veratryl alcohol, Dye decolorization, Carboxylate, Biofuel

## Abstract

**Background:**

Manganese peroxidase is one of the Class II fungal peroxidases that are able to oxidize the low redox potential phenolic lignin compounds. For high redox potential non-phenolic lignin degradation, mediators such as GSH and unsaturated fatty acids are required in the reaction. However, it is not known whether carboxylic acids are a mediator for non-phenolic lignin degradation.

**Results:**

The white rot fungus *Irpex lacteus* is one of the most potent fungi in degradation of lignocellulose and xenobiotics. Two manganese peroxidases (*Il*MnP1 and *Il*MnP2) from *I. lacteus* CD2 were over-expressed in *Escherichia coli* and successfully refolded from inclusion bodies. Both *Il*MnP1 and *Il*MnP2 oxidized the phenolic compounds efficiently. Surprisingly, they could degrade veratryl alcohol, a non-phenolic lignin compound, in a Mn^2+^-dependent fashion. Malonate or oxalate was found to be also essential in this degradation. The oxidation of non-phenolic lignin was further confirmed by analysis of the reaction products using LC–MS/MS. We proved that Mn^2+^ and a certain carboxylate are indispensable in oxidation and that the radicals generated under this condition, specifically superoxide radical, are at least partially involved in lignin oxidative degradation. *Il*MnP1 and *Il*MnP2 can also efficiently decolorize dyes with different structures.

**Conclusions:**

We provide evidence that a carboxylic acid may mediate oxidation of non-phenolic lignin through the action of radicals. MnPs, but not LiP, VP, or DyP, are predominant peroxidases secreted by some white rot fungi such as *I. lacteus* and the selective lignocellulose degrader *Ceriporiopsis subvermispora*. Our finding will help understand how these fungi can utilize MnPs and an excreted organic acid, which is usually a normal metabolite, to efficiently degrade the non-phenolic lignin. The unique properties of *Il*MnP1 and *Il*MnP2 make them good candidates for exploring molecular mechanisms underlying non-phenolic lignin compounds oxidation by MnPs and for applications in lignocellulose degradation and environmental remediation.

**Electronic supplementary material:**

The online version of this article (doi:10.1186/s13068-017-0787-z) contains supplementary material, which is available to authorized users.

## Background

Lignocellulose is a renewable but recalcitrant resource for biofuels and bio-based chemicals [1]. In addition to cellulose and hemicellulose, lignin is one of the major components of lignocellulose. The complex lignin network contains phenolic and non-phenolic lignin sub-structures, with the latter constituting the major part. The white rot fungi (WRF) are regarded to be the best lignocellulose degraders, whose enzymatic systems have hence been an object of extensive studies [2]. WRF produce a range of lignin-modifying enzymes, which include lignin peroxidase (LiP, EC 1.11.1.14), manganese peroxidase [MnP, or Mn(II):H_2_O_2_ oxidoreductase, EC 1.11.1.13], versatile peroxidase (VP, EC 1.11.1.16), laccase (Lac, EC 1.10.3.2), and dye-decolorizing peroxidase (DyP, EC 1.11.1.19) [3]. In addition, free radicals of different types such as hydroxyl radical (OH·), carboxylate anion radical (CO^·−^), and superoxide radical (O_2_^·−^) generated by WRF are also implicated in lignocellulose depolymerization [4]. Among the lignin-modifying enzymes, LiP, VP, and DyP are capable of directly oxidizing non-phenolic lignin model compounds such as veratryl alcohol (VA), whereas MnP and Lac do not have this property [5]. Interestingly, however, MnP and Lac appear to be the most abundant lignin-modifying enzymes for many WRF [3]. This suggests that MnP and Lac may use an alternative mechanism(s) to oxidize the high redox potential non-phenolic lignin moiety.

It is well known that MnP can oxidize Mn^2+^ to Mn^3+^, which forms chelates with organic acids to directly attack the low redox potential phenolic lignin. During this process, unstable free radicals are formed, which tend to disintegrate spontaneously [6]. The chelated Mn^3+^ ions can also react with a certain co-oxidant (or mediator) to generate reactive radicals that can depolymerize the high redox potential non-phenolic lignin. Unsaturated fatty acids (UFA) and their lipid derivatives are such mediators that can be peroxidized to form highly reactive acyl and fatty acid peroxyl radicals acting on the non-phenolic lignin [7–11]. Other mediators such as glutathione (GSH) may also be involved in the formation of thiyl radicals, which are thought to be also involved in the degradation of recalcitrant compounds [12, 13]. However, whether organic acids, particularly those excreted by fungi, are implicated in degradation of high redox potential non-phenolic lignin and xenobiotics has never been clearly demonstrated.

The white rot fungus *Irpex lacteus* has a strong potential in biopretreatment of lignocellulose as well as in biodegradation of xenobiotic compounds. *I. lacteus* appears to produce MnP as the main ligninolytic enzyme under tested conditions [14, 15]. *I. lacteus* CD2 is a strain isolated from Shennong Nature Reserve (Hubei, China) with outstanding capability in degrading lignin and dyes. Although a few MnPs have been purified from the *I. lacteus* cultures, it is not known how these enzymes are involved in destructing lignin and xenobiotics [16, 17]. Herein, we expressed two MnP genes from *I. lacteus* CD2 in *Escherichia coli* and successfully refolded them from inclusion bodies. We showed evidences that MnP-oxidized Mn^3+^ may chelate with a carboxylic acid and form radicals, which are further implicated in degradation of non-phenolic lignin and high redox potential dyes.

## Results and discussion

### Gene cloning and sequence analysis of *Il*MnP1 and *Il*MnP2

The MnPs of *I. lacteus* CD2 have been reported to play an important role in the biological pretreatment of lignocellulose and decolorization of synthetic dyes and even simulated textile wastewater [15]. However, the corresponding mechanism involved in lignin depolymerization and dyes decolorization was unclear. In the present study, two MnP genes (GenBank accession number KX620478 and KX620479), 1684 and 1622 bp, were identified in the genome of *I. lacteus* CD2 (Additional file 1), and their respective cDNAs were successfully obtained from the culture grown on BM medium. The *IlMnP1* and *IlMnP2* were interrupted by 11 introns and 10 introns, giving two open reading frames (ORFs) of 1077 and 1080 bp, respectively (Additional file 1). Deduced *Il*MnP1 and *Il*MnP2 contained 358 and 359 amino acid residues and harbored a signal peptide of 18 and 21 residues, respectively. Similar regulatory elements including TATA box, CAAT motif, CreA- and NIT2-binding sites, putative heat-shock element (HSE), and xenobiotic-responsive element (XRE) were discovered in the upstream region of both genes (Additional file 1). Carbon catabolite repression mediated by CreA or its orthologs was widely found both in ascomycetes [18] and basidiomycetes [19]. The presence of CreA-binding sites implied that the expression of the two MnP genes might be repressed by glucose.

### Refolding and purification of *Il*MnP1 and *Il*MnP2 expressed in *E. coli*

**Fig. 1:**
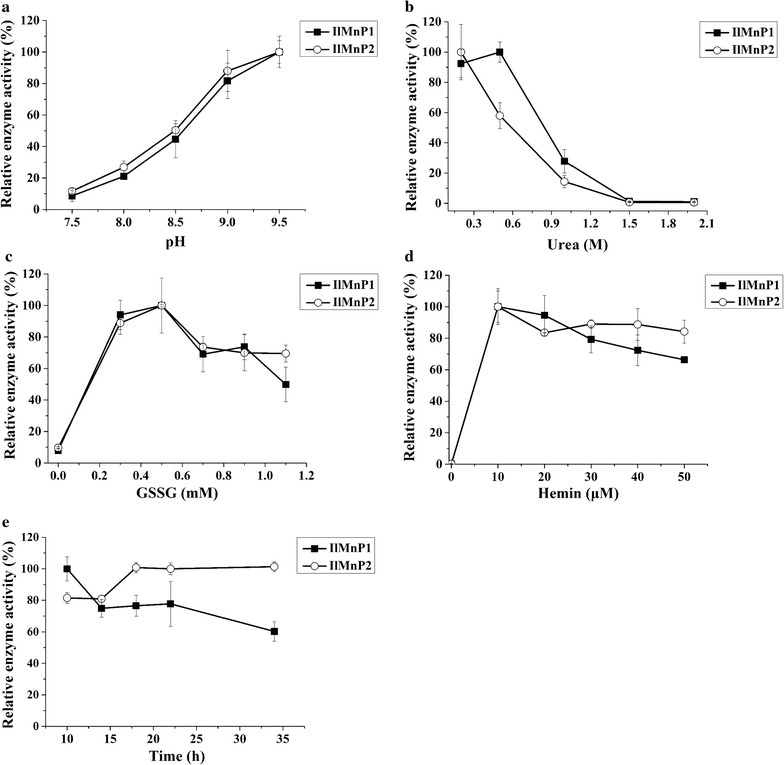
Optimization of the refolding parameters for the recombinant *Il*MnP1 and *Il*MnP2. **a** pH. **b** Urea concentration. **c** GSSG concentration. **d** Hemin concentration. **e** Refolding time. All reactions were performed with 0.1 mg/mL of protein in 50 mM Tris–HCl buffer containing 5 mM Ca^2+^, 0.1 mM EDTA, and 0.1 mM DTT at 15 °C

*Pichia pastoris* and *Trichoderma reesei*, the two popular microbial systems for large-scale production of commercial enzymes, were firstly used as the expressing host but the attempts to express *Il*MnP1 and *Il*MnP2 in these two microbes failed. *E. coli* was then chosen to express these two enzymes. Both *Il*MnP enzymes accumulated exclusively in the inclusion bodies, as had been reported previously for other MnP, VP, and LiP [20, 21]. The inclusion bodies were then solubilized using urea as described previously [20]. Multiple factors including pH, hemin, urea, GSSG, and refolding time are all critical for the successful refolding of peroxidases. By using a fast screening method with 96-well microplates, the optimum pHs for refolding of both enzymes were determined to be pH 9.5 (Fig. 1a), which were the same as that for a VP from *Pleurotus eryngii* [22]. Alkaline pHs were favorable for the formation of thiolate anion, which was essential for the formation of disulfide bridges [22]. Note that both MnPs were predicted to have four disulfide bridges. Different urea concentrations were required for the maximal yield of active *Il*MnP1 (0.2 M) and *Il*MnP2 (0.5 M) in refolding (Fig. 1b). The requirements of MnPs from *I. lacteus* CD2 for urea were much lower than other Class II fungal peroxidases (up to 2 M) [21]. The reducing agents GSSG and DTT were also essential for the formation of disulfide bridges. As shown in Fig. 1c, the optimal GSSG/DTT ratios for the MnPs were 5:1 (0.5 mM GSSG versus 0.1 mM DTT). Although hemin was not necessary for the refolding of other Class II fungal peroxidase or the horseradish peroxidase, it was required for the refolding of *Il*MnP1 and *Il*MnP2 at an optimal concentration of 10 μM (Fig. 1d). Over the time course, the refolding of *Il*MnP2 significantly increased from 10 to 20 h to reach a plateau, while that of *Il*MnP1 decreased instead from 10 h (Fig. 1e).

**Fig. 2:**
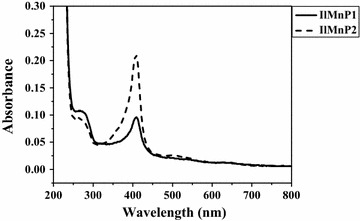
Purified recombinant *Il*MnP1 and *Il*MnP2 in the 20 mM malonate buffer (pH 5.0) as analyzed by UV–visible spectroscopy at the spectrums ranging from 230 to 800 nm

Large-scale refolding of the *Il*MnP1 and *Il*MnP2 was conducted under the optimized conditions (0.5 mM GSSG, 0.1 mM DTT, 10 μM hemin, 5 mM CaCl_2_, and 0.1 mg/mL protein, 0.5 M urea for *Il*MnP1 or 0.2 M urea for *Il*MnP2, pH 9.5) for 10 h at 15 °C. The refolded proteins were further purified by anion exchange. Finally, yields of 28.2 mg and 13.3 mg of functional *Il*MnP1 and *Il*MnP2, respectively, per liter culture were obtained. Both enzymes showed a single band on SDS-PAGE gels, corresponding to the calculated molecular masses (Additional file 2). Moreover, the enzymes had an absorbance peak at 409 nm (Fig. 2), indicating that each MnP harbors a heme group [23]. The Rz (A_407_/A_280_) ratios of *Il*MnP1 and *Il*MnP2 were 1.0 and 2.4, respectively.

### Optimal pH and temperature of *Il*MnP1 and *Il*MnP2

The optimal pHs of recombinant *Il*MnP1 and *Il*MnP2 were both pH 4.0 (Additional file 3a). When the pH was above 6.5, no activity was detected for both enzymes. This character was similar to that of native MnPs from *I. lacteus* strains CD2 (pH 3.0–6.0) and Fr. 238 (pH 3.0–7.6) and other fungi, which are all acidic MnPs (Table 1). The two MnPs varied in pH stability (Additional file 3b). At neutral pH, the *Il*MnP2 retained much more residual activity than *Il*MnP1. Interestingly, most native or recombinant MnPs from *I. lacteus* ever reported exhibit remarkable stability at neutral pH, while one MnP from *Phanerochaete chrysosporium* was inactive at near neutral pH (6.5) [24]. The optimal temperatures of *Il*MnP1 and *Il*MnP2 were both 60 °C (Additional file 3c). However, at 60 °C both enzymes quickly lost their activity within 10 min (Additional file 3d). The thermostability of the two recombinant *Il*MnPs were not as good as a natively purified MnP from *I. lacteus* CD2: the native *I. lacteus* CD2-MnP retained 93.2% of the initial activity after 1 h of incubation at 40 °C. At this temperature, *Il*MnP2 retained 80.5% activity, while *Il*MnP1 had only 13.1% left after 1 h of incubation [15]. This weakness in thermostability might be ascribed to the lack of glycosylation during heterologous expression in *E. coli* [25].

**Table 1:** Comparison of the biochemical properties of recombinant *Il*MnP1 and *Il*MnP2 with other MnPs

Enzyme	Organism	MW (kDa)	p*I*	pH optimum	Temperature optimum	*K*_m_ for Mn^2+^ (μM)	Reference
*Il*MnP1	*I. lacteus* CD2	38	–	4.0	60	193.8	This study
*Il*MnP2	*I. lacteus* CD2	38	–	4.0	60	152.2	This study
CD2-MnP	*I. lacteus* CD2	42	–	4.5	70	49	[15]
MnP	*I. lacteus* CCBAS238	37	3.55	5.5	60	31	[17]
MnP	*I. lacteus*	47	–	6.0	50–60	22	[38]
MnP	*I. lacteus* Fr. 238	37	4.8	5.0	–	47	[16]
rMnP	*I. lacteus* F17	43	–	6.5	25	95	[23]
MnP	*Bjerkandera adusta*	43	3.55	5.0	–	17	[39]
MnP	*Schizophyllum* sp. F17	49	–	6.8	35	35	[40]
MNP1	*Agrocybe praecox*	42	6.4	–	–	17	[41]
MNP	*P. chrysosporium*	45	–	4.5	30	–	[24]

### Biochemical analysis of *Il*MnP1 and *Il*MnP2 on Mn^2+^ and phenolic lignin model compounds

The *K*_m_ values of *Il*MnP1 and *Il*MnP2 for Mn^2+^ were 193.8 and 152.2 μM, respectively, higher than those of native MnPs (17–49 μM) (Table 1). The *k*_cat_ values of *Il*MnP1 and *Il*MnP2 were 7.1 and 6.6 s^−1^, respectively. The structures and maximal absorbance wavelengths of the substrates (phenolic, non-phenolic lignin model compounds, and dyes) used in this study are listed in Table 2. *Il*MnP1 and *Il*MnP2 could oxidize two phenolic substrates DMP and guaiacol as well as the substrate ABTS, with the specific activities significantly higher in the presence of Mn^2+^ (Table 3). Although *Il*MnP1 and *Il*MnP2 can directly attack phenolic lignin model compounds, both enzymes exhibited significant Mn^2+^-dependent activity, which was commonly found in MnPs. For example, the *k*_cat_ of the native MnP from *I. lacteus* CCBAS238 on DMP in the presence of Mn^2+^ was 15.7 s^−1^, 26.2-fold higher than that (0.6 s^−1^) without Mn^2+^ [16]. The oxidation of phenolic substrates by MnPs was thought to be through one-electron oxidation involving the chelated Mn^3+^ ions [6].

**Table 2:** Lignin model compounds (LMC) and synthetic dyes used in this work

Class	Substrate	Structure	*λ*_max_ (nm)
LMC: phenolic	DMP		470
	Guaiacol		465
LMC: non-phenolic	VA		310
Other	ABTS		420
Dye: monoazo	Remazol brilliant violet 5R		556
Dye: disazo	Reactive back 5		596
Dye: anthraquinone	Remazol brilliant blue R		600
Dye: indigo	Indigo carmine		610
Dye: triphenylmethane	Methyl green		640

**Table 3:** Substrate specificities of recombinant *I. lacteus* CD2 manganese peroxidases

Substrate^a^	*E*_max_ (M^−1^ cm^−1^)	Wavelength (nm)	*Il*MnP1 (U/L)		*Il*MnP2 (U/L)	
			Mn^2+^ present	Mn^2+^ absent	Mn^2+^ present	Mn^2+^ absent
ABTS	36,000	420	920 ± 43	480 ± 5	933 ± 29	160 ± 3
DMP	27,500	470	380 ± 6	14 ± 1	436 ± 10	4 ± 0
Guaiacol	12,100	465	142 ± 10	4 ± 0	234 ± 5	2 ± 0

^a^The concentration of each substrate was 1 mM

### Degradation of a non-phenolic lignin model compound by *Il*MnP1 and *Il*MnP2

**Fig. 3:**
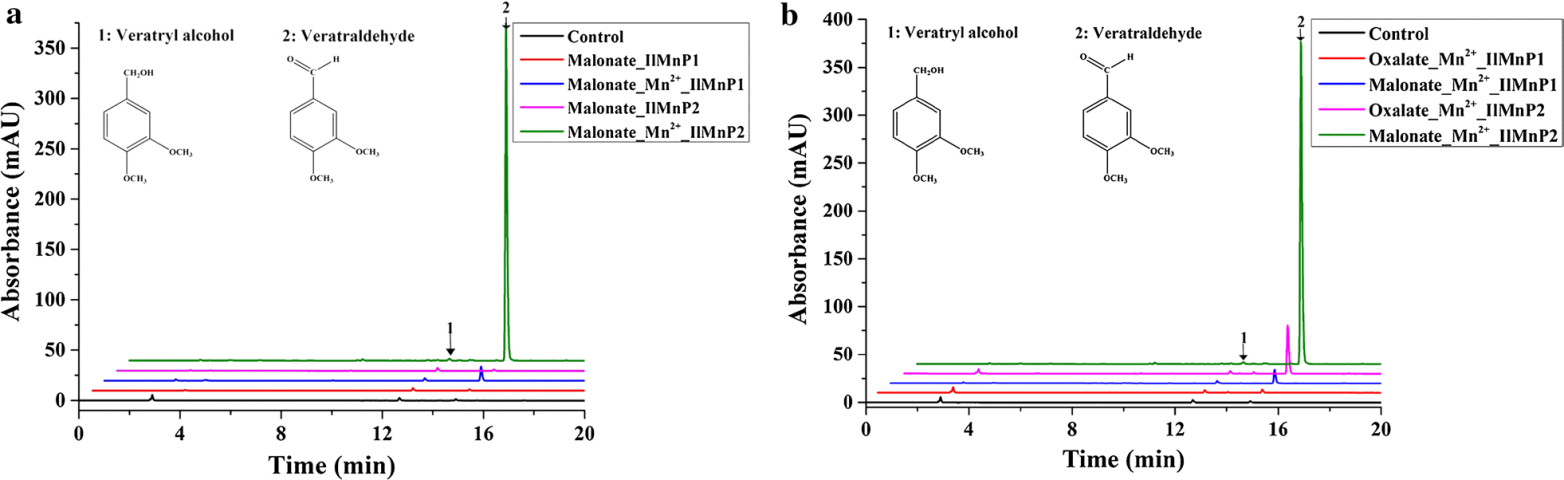
Oxidation of the non-phenolic lignin model compound veratryl alcohol by 0.5 U/mL *Il*MnP1 and *Il*MnP2 at 30 °C for 48 h. **a** VA was treated by the two *Il*MnPs in the malonate buffer (50 mM, pH 5.0) with or without 1 mM Mn^2+^. **b** VA was treated by the two *Il*MnPs in the malonate and oxalate buffer (50 mM, pH 5.0) with 1 mM Mn^2+^. *Control* VA was treated without any enzyme in the malonate buffer (pH 5.0) with 1 mM Mn^2+^

Surprisingly, the two MnPs could also oxidize the non-phenolic substrate VA albeit only in the presence of Mn^2+^ (Fig. 3a). No MnP has been reported previously to have VA-oxidizing ability, which was thought to be a unique catalytic feature of high redox potential peroxidases such as LiP and VP. These VA-oxidizing enzymes commonly have a tryptophan residue involved in VA binding near the heme-binding site [26]. Interestingly, neither *Il*MnP1 nor *Il*MnP2 bears such a characteristic tryptophan (aspartate for *IlI*MnP1 and alanine for *IlI*MnP2 at the corresponding position instead, Additional file 4), excluding the possibility that *Il*MnP1 and *Il*MnP2 are LiPs or VPs. The unusual catalysis of VA suggested that another mechanism must be involved in oxidation of VA by *Il*MnP1 and *Il*MnP2.

**Fig. 4:**
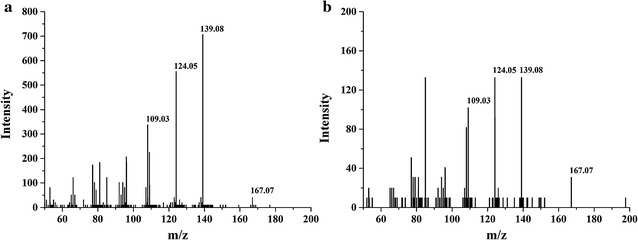
LC–MS/MS spectra for veratraldehyde (standard, **a**) and its reaction products by *Il*MnP2 (50 mM pH 5.0 malonate buffer, 30 °C for 48 h, with 1 mM Mn^2+^, **b**)

In order to confirm that *Il*MnP1 and *Il*MnP2 could oxidize the non-phenolic lignin model compound VA, the reaction products were further analyzed by HPLC as well as LC–MS/MS. A peak corresponding to veratraldehyde was clearly detected in reactions in the presence of *Il*MnP1 and *Il*MnP2, Mn^2+^, and malonate (Fig. 3a) or oxalate (Fig. 3b) but not with acetate, citrate, lactate, or succinate (Additional file 5). Figure 4 is a representative result of the LC–MS/MS analysis with the positive ionization mode, which was conducted with the *Il*MnP2 reaction product (*Il*MnP1 appeared to have the same pattern of VA oxidation but a lower peak in HPLC thus not included in MS/MS analysis). The veratraldehyde standard has a molecular weight of 166, thus producing daughter ions of 139 [M-28+H]^+^, 124 [M-43+H]^+^, and 109 [M-58+H]^+^ (Fig. 4a). These ions could also be observed in the MS/MS analysis of *Il*MnP2-VA reaction products, confirming that *Il*MnP2 could indeed convert VA into veratraldehyde (Fig. 4) [27].

The catalysis depends on the presence of both Mn^2+^ and a specific carboxylic acid since VA was not degraded when Mn^2+^ was absent (Fig. 3a) or if malonate was replaced by acetate, citrate, lactate, or succinate (Additional file 5). Acetate and succinate could not form complexes with Mn^3+^ [28]; therefore, no oxidation product was observed in the acetate or succinate buffer. Mn^3+^ can form chelates with the rest four organic acids. Mn^3+^-lactate/tartrate complexes were reported to react rapidly with H_2_O_2_ to generate O_2_ [28]. Therefore, we hypothesized that Mn^3+^-lactate/citrate complexes was more apt to react with H_2_O_2_ than malonate/oxalate. However, this phenomenon was not detected for the malonate or oxalate buffer [28]. Moreover, it was reported that the carbon-centered radical and superoxide radical were generated from the oxidation of malonate/oxalate by Mn^3+^ [29, 30]. The VA-oxidizing activities of *Il*MnP1 and *Il*MnP2 in malonate were higher than those in oxalate (Fig. 3b), and oxalate was also reported to be ineffective in supporting VA oxidation by MnP from *Lentinus edodes* [31]. These clearly indicated that both Mn^2+^ and the carboxylate play an indispensable role in degrading the non-phenolic lignin model compound by *Il*MnP1 and *Il*MnP2.

**Fig. 5:**
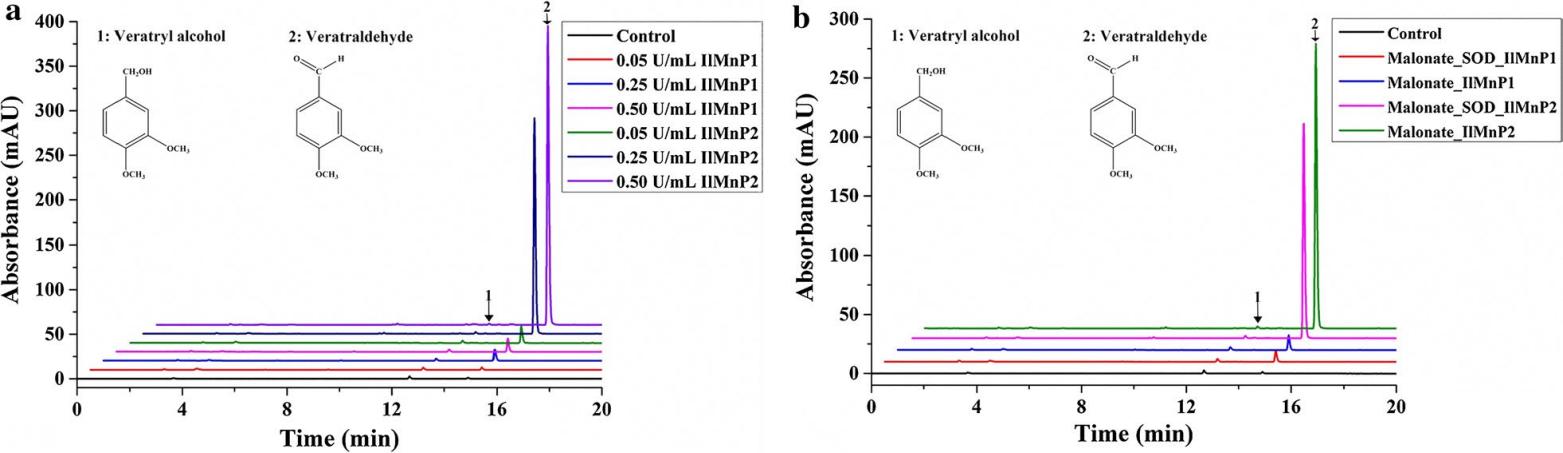
The effect of enzyme loading (**a**) and superoxide dismutase (**b**) on the oxidation of veratryl alcohol by *Il*MnP1 and *Il*MnP2 in the malonate buffer (50 mM, pH 5.0) at 30 °C for 48 h with 1 mM Mn^2+^. *Control* VA was treated without any enzyme in the malonate buffer (50 mM, pH 5.0) with 1 mM Mn^2+^

GSH and UFA have been reported to mediate oxidation of non-phenolic lignin compounds by MnPs through generation of highly active thiyl and fatty acid peroxyl radicals, respectively [6, 13]. Note that carboxylic acids can also be oxidized by chelated Mn^3+^ to generate radicals [6, 29]. We also noticed that the extent of VA oxidation by *Il*MnP1 and *Il*MnP2 improved when enzyme loading increased from 0.05 to 0.25 and then to 0.5 U/mL, particularly for *Il*MnP2 (Fig. 5a). Besides, the oxidation product veratraldehyde steadily increased with a linear relationship to the concentrations of VA (Additional file 6). However, whether the radicals generated in the MnP-Mn^2+^-carboxylic acids system are able to attack the high redox potential non-phenolic lignin is not known from previous literature studies. Since Mn^2+^ and a certain carboxylate (malonate and oxalate) are the two necessary components needed for *Il*MnP1 and *Il*MnP2 to degrade non-phenolic lignin compounds, it is now reasonable to infer that the ability of *Il*MnP1 and *Il*MnP2 to oxidize VA was actually through the action of radicals, which were generated by the reactions of MnP-oxidized Mn^3+^ with malonate or oxalate. To gain some insights of VA oxidation by the two *Il*MnPs, the reactions in the malonate buffer were performed in the presence or absence of SOD (at a final concentration of 3000 U/mL), which is a commonly used scavenger for superoxide radical [32]. SOD had no inhibitory effect on the formation of Mn^3+^ (for *Il*MnP1, 1048 and 1053 U/L in absence and presence of SOD, respectively; for *Il*MnP2, 965 and 1000 U/L in absence and presence of SOD, respectively) but partially inhibited the oxidation of VA by *Il*MnP1 (by 25.3%) and *Il*MnP2 (by 23.9%) (Fig. 5b). This indicated that the superoxide radical is at least partially responsible for VA oxidation by the two MnPs in presence of a certain carboxylic acid. Interestingly, both malonate and oxalate are known to be acids excreted by saprophytic fungi including *I. lacteus* and *P. chrysosporium* [8, 28]. Our results suggest that *I. lacteus* may use its MnPs with a particular organic acid(s) it excretes to co-operate in degrading the more recalcitrant lignin.

Interestingly, while the pH optimum for LiP, VP, and DyP in oxidizing VA is pH 3 or lower [5, 26, 33], *Il*MnP1 and *Il*MnP2 exhibited VA-oxidizing ability at pH 5, which is also optimal for the mostly used *T. reesei* cellulases [34] and similar to those of many other acidic plant cell wall polysaccharides degrading enzymes [35, 36]. MnP, in the presence of Mn^2+^ and a carboxylic acid mediator such as malonate, may hence be used to formulate enzyme cocktails with cellulase and hemicellulase to simultaneously deconstruct lignin, cellulose, and hemicellulose. Besides the application potential in lignocellulose degradation, *Il*MnP1 and *Il*MnP2 may also serve as good candidates for further investigating the molecular mechanisms underlying non-phenolic lignin depolymerization by MnPs.

### Application potential of *Il*MnP1 and *Il*MnP2 in decolorizing dyes with different structures

**Fig. 6:**
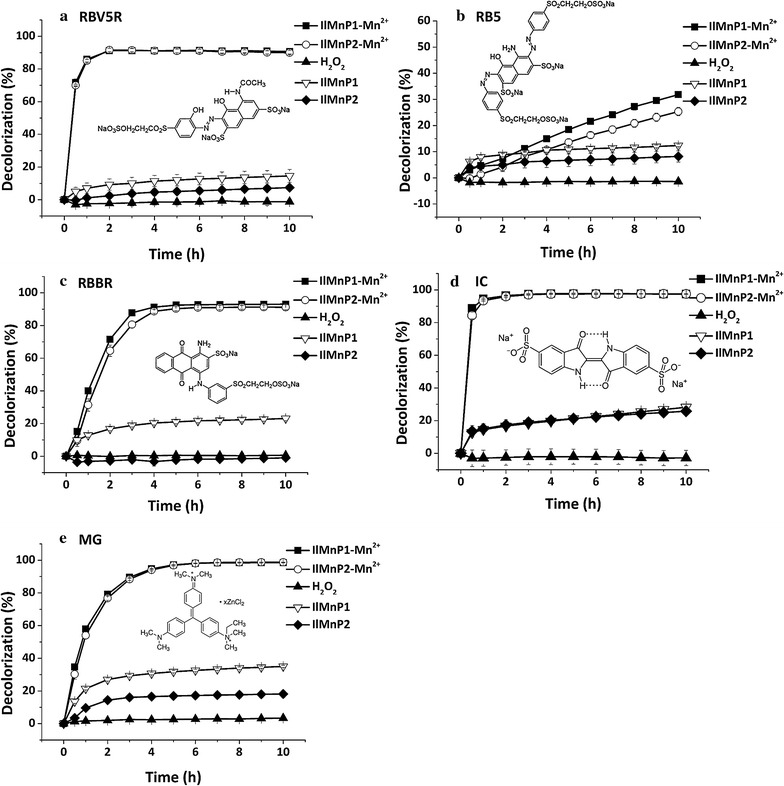
Decolorization of dyes with different structures by recombinant *Il*MnP1 and *Il*MnP2. **a** RBV5R. **b** RB5. **c** RBBR. **d** IC. **e** MG. The reactions were carried out at 30 °C containing 50 mM malonate buffer (pH 5.0), 0.1 mM H_2_O_2_, 0.25 U/mL *Il*MnP1 or *Il*MnP2, and 50 mg/L of dye, with or without 1 mM Mn^2+^

*Il*MnP1 and *Il*MnP2 are capable of directly or indirectly degrading phenolic and non-phenolic lignin compounds with varying structures, which enlightens us to explore if they also have the ability to decolorize dyes for environmental remediation. A purified MnP from *I. lacteus* CD2 has been reported to be able to efficiently decolorize different types of dyes [15]. *Il*MnP1 and *Il*MnP2 also exhibited strong ability to decolorize a broad range of dyes including the azo dyes (RBV5R and RB5), anthraquinone dye (RBBR), indigo dye (IC), and triphenylmethane (MG) in the presence of Mn^2+^ and malonate (Fig. 5). The decolorization of RBV5R and IC was the fastest: above 85% of the dyes (50 mg/L) could be decolorized by the enzymes within 1 h (Fig. 6a, d). In contrast, the degradation of RBBR and MG was much slower; more than 90% of the dyes could be decolorized after 5 h of incubation (Fig. 6c, e). Nonetheless, the decolorization of MG by *Il*MnP1 and *Il*MnP2 was much more effective than that by the purified MnP from *I. lacteus* CD2 (32% decolorized after 36 h of incubation [15]). The degradation of RB5 was the slowest: 31.9 and 25.4% were decolorized by *Il*MnP1 and *Il*MnP2, respectively, within 10 h (Fig. 6b). RB5 is considered a specific substrate for VP but not oxidized by the MnP of *P. chrysosporium* in a tartrate buffer [37]. Note that the rate of dye decolorization was significantly reduced in the absence of Mn^2+^ for all dyes. These together support the notion that, like lignin degradation, Mn^2+^ and a specific carboxylate such as malonate are important constituents for efficient dye decolorization by the two MnPs from *I. lacteus* CD2.

## Conclusions

In this study, two manganese peroxidase genes were cloned from the white rot fungus *I. lacteus* CD2. By optimizing a variety of parameters, the *E. coli*-expressed *Il*MnP1 and *Il*MnP2 were successfully refolded from inclusion bodies. The recombinant *Il*MnP1 and *Il*MnP2 could oxidize a series of phenolic and even non-phenolic lignin model compounds substrate VA. Mn^2+^ and a certain carboxylate (malonate or oxalate) are the two indispensable components in enzymatic degradation of the non-phenolic lignin. It is proposed that radicals such as superoxide radical formed in this carboxylate buffer system are at least partially involved in degrading these high redox potential lignin compounds. Besides, *Il*MnP1 and *Il*MnP2 could also decolorize dyes of four different types, whose efficiency also depended on the presence of Mn^2+^. In summary, we demonstrated that the degradation of non-phenolic lignin by MnP is not restricted to GSH or UFA mediators but can expand to carboxylic acids, which are excreted by fungi as a normal metabolite. The properties of *Il*MnP1 and *Il*MnP2 make them ideal candidates for exploring molecular mechanisms underlying lignin deconstruction by MnPs and potential players in formulating efficient enzyme cocktails for lignocellulose degradation and dye decolorization.

## Methods

### Strain and substrates

*Irpex lacteus* CD2 was isolated from Shennong Nature Reserve (Hubei province, China) and preserved in the Institute of Environment & Resource Microbiology, Huazhong University of Science & Technology, Wuhan, China. *I. lacteus* CD2 was maintained at 4 °C on potato-dextrose agar (PDA) plate. Substrates 2,2′-azino-bis(3-ethylbenzothiazoline-6-sulfonic acid) (ABTS), 2,6-dimethylphenol (DMP), veratryl alcohol (VA), guaiacol, and dyes with various structures including remazol brilliant violet 5R (RBV5R), reactive black 5 (RB5), remazol brilliant blue R (RBBR), methyl green (MG), and indigo carmine (IC) were purchased from Sigma-Aldrich (St. Louis, MO). The superoxide dismutase (SOD) was purchased from Solarbio (Beijing, China). The structures for the substrates, synthetic dyes, and non-phenolic lignin model compound are listed in Table 2.

### Cloning and expression of *IlMnP1* and *IlMnP2*

*Irpex lacteus* CD2 was grown for 5 days in the basal liquid medium [15]. Total RNA was extracted using the TRIZOL reagent (Invitrogen, Waltham, MA) according to the manufacturer’s instructions. The first strand cDNA was synthesized from the total RNA using the TransScript One-Step gDNA Removal and cDNA Synthesis Supermix with oligo (dT) (TransGen). Based on the 5′- and 3′-end sequences of the *Il*MnP1 and *Il*MnP2 structural genes, the MnP genes devoid of the sequences encoding the signal peptides were amplified with gene-specific primers (as shown in Additional file 7). The PCR products were T-A ligated into pEASY-T3 (TransGen) and then transformed into the *E. coli* Trans1-T1 to obtain pEASY-T3-*Il*MnP1 and pEASY-T3-*Il*MnP2.

The recombinant plasmids pEASY-T3-*Il*MnP1 and pEASY-T3-*Il*MnP2 were double-digested with *Bam*HI/*Not*I and *Bam*HI/*Xho*I, respectively, gel purified, and ligated into the pre-digested pET-28a(+) to obtain pET28a-*Il*MnP1 and pET28a-*Il*MnP2, and individually transformed into *E. coli* BL21 (DE3) competent cells. The cells harboring pET28a-*Il*MnP1 or pET28a-*Il*MnP2 were pre-cultured in LB medium supplemented with 50 μg/mL of kanamycin at 37 °C overnight with shaking at 200 rpm and used as the inocula of 200 mL LB medium. The cultures were grown at 37 °C for 3 h, followed by the addition of isopropyl-β-d-thiogalactoside (IPTG) to a final concentration of 1 mM for 4-h induction.

After induction, the cells were harvested by centrifugation. The pellets were re-suspended in 50 mM Tris–HCl, 10 mM EDTA, and 5 mM DTT (pH 8.0). Lysozyme (Amresco, Solon, OH) was then added to a final concentration of 2 mg/mL and the cells were incubated on ice for 1 h. Then, 20 μL of DNase I (TransGen) was added and the incubation was continued on ice for 30 min. Subsequently, the cells were centrifuged at 12,000*g* for 30 min at 4 °C. No MnP activity could be detected from the supernatants. The cell debris was washed with 20 mM Tris–HCl, 1 mM EDTA, and 5 mM DTT (pH 8.0) twice, followed by incubation in 50 mM Tris–HCl, 8 M urea, 1 mM EDTA, and 1 mM DTT (pH 8.0) on ice for 1 h.

To optimize the parameters for recovering active enzyme from inclusion bodies, the refolding was performed in various conditions in a 200 μL volume using 96-well plates at 15 °C. A range of parameters including concentrations of urea, GSSG, and hemin and pH were investigated, while the concentrations of enzyme, EDTA, and DTT were kept constant during the refolding. The efficiency of refolding was indicated by the MnP activity. Based on the fast plate-screening result, large-scale refolding of MnPs was performed using the respective optimum parameters. After refolding, the crude enzymes were centrifuged at 12,000*g* for 10 min at 4 °C and the insoluble fractions were discarded. The supernatants containing the refolded MnP were concentrated through a 10 kDa cut-off centrifuge filter, followed by dialysis against 20 mM phosphate buffer, pH 6.0. The crude enzymes were further purified by a HiTrap Q HP anion exchange column (GE Health, Fairfield, CT) pre-equilibrated with the same phosphate buffer. The proteins were eluted with a linear gradient of 0–1.0 M NaCl, and fractions containing active enzymes were pooled.

### Biochemical characterization of *Il*MnP1 and *Il*MnP2

The refolded *Il*MnP1 and *Il*MnP2 were first subjected to UV–visible spectroscopic analysis in the range of 230–800 nm in the 20 mM malonate buffer (pH 5.0). The MnP activity was measured by monitoring the oxidation of ABTS (*ε*_420_ = 36,000 M^−1^ cm^−1^) at 420 nm, in a buffer containing 50 mM malonate, 1 mM ABTS, 1 mM MnSO_4_, and 0.1 mM H_2_O_2_ (pH 5.0 and 25 °C). For the Mn^2+^-independent activity assay, MnSO_4_ was omitted. One unit (U) of enzyme activity was defined as the amount of enzyme that oxidizes 1 μmol of ABTS per min at 25 °C [30]. For kinetic studies, the reactions were performed in the 50 mM malonate buffer (pH 5.0) at 25 °C using 10–4000 μM Mn^2+^ (in the presence of 0.1 mM H_2_O_2_) as the substrate by monitoring the formation of Mn^3+^-malonate complexes (*ε*_270_ = 11,590 M^−1^ cm^−1^) at 270 nm [15]. The non-linear least square fitting method was used to calculate the *K*_m_, *k*_cat_, and *k*_cat_/*K*_m_ parameters of the recombinant *Il*MnP1 and *Il*MnP2 using the GraphPad Prism 5 software.

To determine the pH optimum, the MnP activity on ABTS was determined in the 20 mM malonate buffer at a pH ranging from 3.0 to 7.0 at 25 °C. For temperature optimum, the enzymatic activity was measured in the 20 mM malonate buffer (pH 5.0) at a temperature from 20 to 80 °C. To evaluate the pH stability, *Il*MnP1 and *Il*MnP2 were individually incubated at different pH levels (3.0–7.0) for 1 h, and the residual activities were assayed as described above. For thermostability, *Il*MnP1 and *Il*MnP2 were incubated at 40–60 °C for 1 h with samples taken for activity measurement periodically. The residual activities were measured at its optimum pH and temperature.

The substrates specificities of *Il*MnP1 and *Il*MnP2 were studied for the oxidation of four different substrates ABTS, DMP, guaiacol, and VA in 50 mM pH 5.0 malonate and 0.1 mM H_2_O_2_ with or without 1 mM MnSO_4_. Activities were calculated using absorption coefficients at the corresponding wavelengths.

### Oxidation of non-phenolic lignin model compounds by *Il*MnP1 and *Il*MnP2

VA was used in evaluating the abilities of *Il*MnP1 and *Il*MnP2 for degradation of the non-phenolic lignin compound. The degradation of VA was performed in 50 mM malonate buffer (pH 5.0) containing 1 mM VA, 1 mM MnSO_4_, 0.1 mM H_2_O_2_, and 0.5 U/mL *Il*MnP1 or *Il*MnP2. In some reactions, the malonate buffer was changed to another carboxylate (acetate, oxalate, citrate, lactate, or succinate) buffer (pH 5.0) or MnSO_4_ was omitted from the reaction. The effect of VA concentration (0.05–1 mM) on oxidation was analyzed in the malonate buffer (pH 5.0) for 48 h with 0.5 U/mL *Il*MnP1 or *Il*MnP2 in the presence of 1 mM Mn^2+^. The effect of enzymes loading and superoxide dismutase (3000 U/mL) on VA oxidation by *Il*MnP1 or *Il*MnP2 was analyzed in the malonate buffer (pH 5.0) for 48 h in the presence of 1 mM Mn^2+^. The ability of Mn^3+^ formation was evaluated by the oxidation of ABTS. The reaction proceeded at 30 °C for 48 h, and then the reaction products were analyzed by HPLC using a reversed phase C_18_-column (Eclips XDB-C_18_, 4.6 mm × 250 mm, 5 μm). The elution condition was 0% Acetonitrile (ACN), 4 min; 0–60% ACN, 10 min; 60–100% ACN, 1 min; and 100% ACN, 5 min at a flow rate of 0.8 mL/min. The elution peaks were monitored at 310 nm. In order to confirm veratraldehyde as the oxidation product, LC–MS was also performed by coupling a Nexera UHPLC system to an AB-SCIEX 5600 Triple TOF mass spectrometer in positive and high-sensitivity mode. For LC analysis, the column, mobile phase, detection, and flow rate were identical to those for HPLC analysis described above. The elution program was as follows: 0–60% ACN, 10 min; 60–100% ACN, 1 min; and 100% ACN, 5 min. For MS analysis, the parameters were set as ion source gases GS1, GS2, and curtain gas were 55, 55, and 35 psi, respectively, temperature was 600 °C, and ion spray voltage floating was at 5500 V.

### Decolorization of dyes by *Il*MnP1 and *Il*MnP2

Five dyes of different structures were used to evaluate the decolorization capability of *Il*MnP1 and *Il*MnP2. The reactions were carried out at 30 °C in a total volume of 200 μL containing 50 mM malonate buffer (pH 5.0), 0.1 mM H_2_O_2_, 0.25 U/mL *Il*MnP1 or *Il*MnP2, and 50 mg/L of dye, with or without 1 mM Mn^2+^. During the incubation, the color changes were periodically detected by measuring the optical density (OD) at 556 nm for RBV5R, 596 nm for RB5, 600 nm for RBBR, 610 nm for IC, and 640 nm for MG. The rate of decolorization was then calculated using the following formula: decolorization (%) = [(*A*_i_ − *A*_t_)/*A*_i_]×100, where *A*_i_ and *A*_t_ are the absorbance at the initial and given stages.

## Additional files

**Additional file 1.** The nucleotide and deduced amino acid sequences of the manganese peroxidase isoenzymes *Il*MnP1 (a) and *Il*MnP2 (b) of *I. lacteus* CD2. The signal peptide of the two manganese peroxidases was shown in red. The putative Cis-acting elements in the regulatory region are underlined. CreA: CreA-binding sites; XRE: xenobiotic-responsive elements; NIT2: NIT2 transcription factor consensus binding sequences; HSE: heat shock element.

**Additional file 2.** Purified recombinant *Il*MnP1 and *Il*MnP2 as analyzed by SDS-PAGE. Lanes: M, the protein molecular mass marker; 1, the purified *Il*MnP1; 2, the purified *Il*MnP2.

**Additional file 3.** Effect of pH and temperature on the activity and stability of *Il*MnP1 and *Il*MnP2. (a) The pH-activity profiles. The activities at the pH optima were set as 100%. (b) The pH-stability profiles. The initial MnP activities before treatment were set as 100%. (c) The temperature-activity profiles. The activities at the temperature optima were set as 100%. (d) The temperature-activity profiles. The initial MnP activities before heat treatment were set as 100%.

**Additional file 4.** Amino acid sequence alignment of *Il*MnP1 and *Il*MnP2 with selected MnPs, VPs, and LiPs. Inverted triangle: the cysteines that might form disulfide bridges; diamond: the structural Ca^2+^-binding residues; triangle: the active site histidine residues; hexagon: the acid residues forming the Mn^2+^ oxidation site; square: the tryptophan responsible for aromatic substrate oxidation. The GenBank accession numbers for these enzymes were: *Il*MnP1, KX620478; *Il*MnP2, KX620479; *Po*MnP2, KDQ32034.1; *Po*MnP4, 4BM1; *Po*MnP5, KDQ27903.1; *Po*MnP6, KDQ28248.1; *Pc*MnP-H4, P19136.1; *Pe*VP2, 2BOQ; *Pc*LiP-H2, P11542.2; *Pc*LiP-H8, AAB00798.1.

**Additional file 5.** The non-phenolic lignin model compound veratryl alcohol was not oxidized by either *Il*MnP1 or *Il*MnP2 as analyzed by HPLC. The enzymes (0.5 U/mL *Il*MnP1 and *Il*MnP2, respectively) were incubated with VA in the acetate, citrate, lactate, or succinate buffer (50 mM, pH 5.0) with 1 mM Mn^2+^ at 30 °C for 48 h.

**Additional file 6.** The product veratraldehyde steadily increased with a linear relationship to the concentrations of VA when oxidized by 0.5 U/mL *Il*MnP1 (a) or *Il*MnP2 (b). The reaction systems contained the malonate buffer (pH 5.0) and were incubated for 48 h in presence of 1 mM Mn^2+^.

**Additional file 7.** Primers used in this study.

## Abbreviations

WRF: white rot fungi
LiP: lignin peroxidase
MnP: manganese peroxidase
VP: versatile peroxidase
Lac: laccase
DyP: dye-decolorizing peroxidase
UFA: unsaturated fatty acids
GSH: glutathione
ORFs: open reading frames
HSE: heat-shock element
XRE: xenobiotic-responsive element
PDA: potato-dextrose agar
ABTS: 2,2′-azino-bis (3-ethylbenzothiazoline-6-sulfonic acid)
DMP: 2,6-dimethylphenol
VA: veratryl alcohol
RBV5R: remazol brilliant violet 5R
RB5: reactive black 5
RBBR: remazol brilliant blue R
MG: methyl green
IC: indigo carmine
SOD: superoxide dismutase
IPTG: isopropyl-β-d-thiogalactoside
ACN: acetonitrile
