# Supplementary material for: Oxidation of a non-phenolic lignin model compound by two Irpex lacteus manganese peroxidases: evidence for implication of carboxylate and radicals
Source: Biotechnol Biofuels. 2017 Apr 21;10:103. doi: 10.1186/s13068-017-0787-z (PMC5399396; doi:10.1186/s13068-017-0787-z)
Supplement: Supplementary file 1 — Additional file 1. The nucleotide and deduced amino acid sequences of the manganese peroxidase isoenzymes IlMnP1 (a) and IlMnP2 (b) of I. lacteus CD2. The signal peptide of the two manganese peroxidases was shown in red. The putative Cis-acting elements in the regulatory region are underlined. CreA: CreA-binding sites; XRE: xenobiotic-responsive elements; NIT2: NIT2 transcription factor consensus binding sequences; HSE: heat shock element. [file 13068_2017_787_MOESM1_ESM.doc]

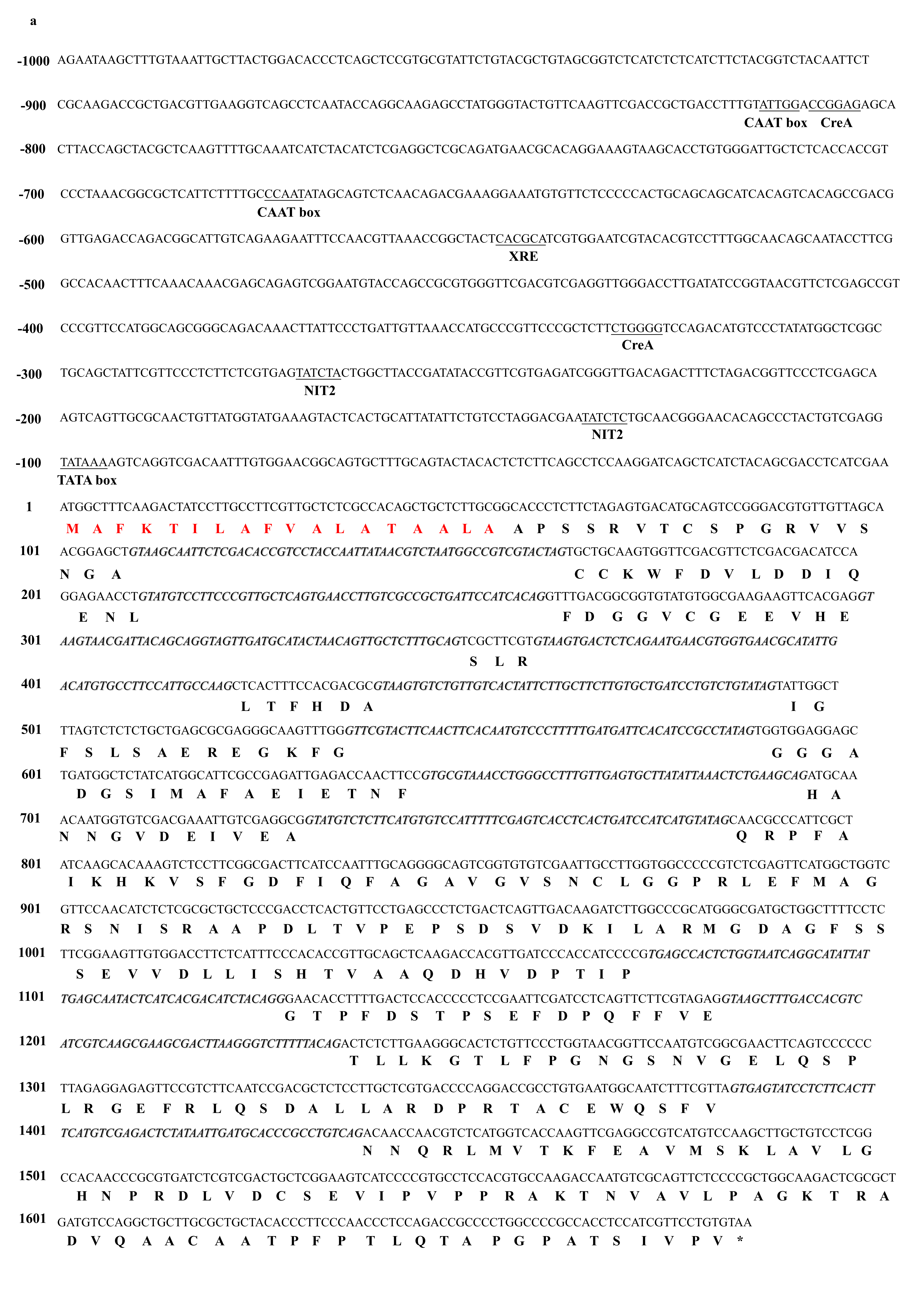

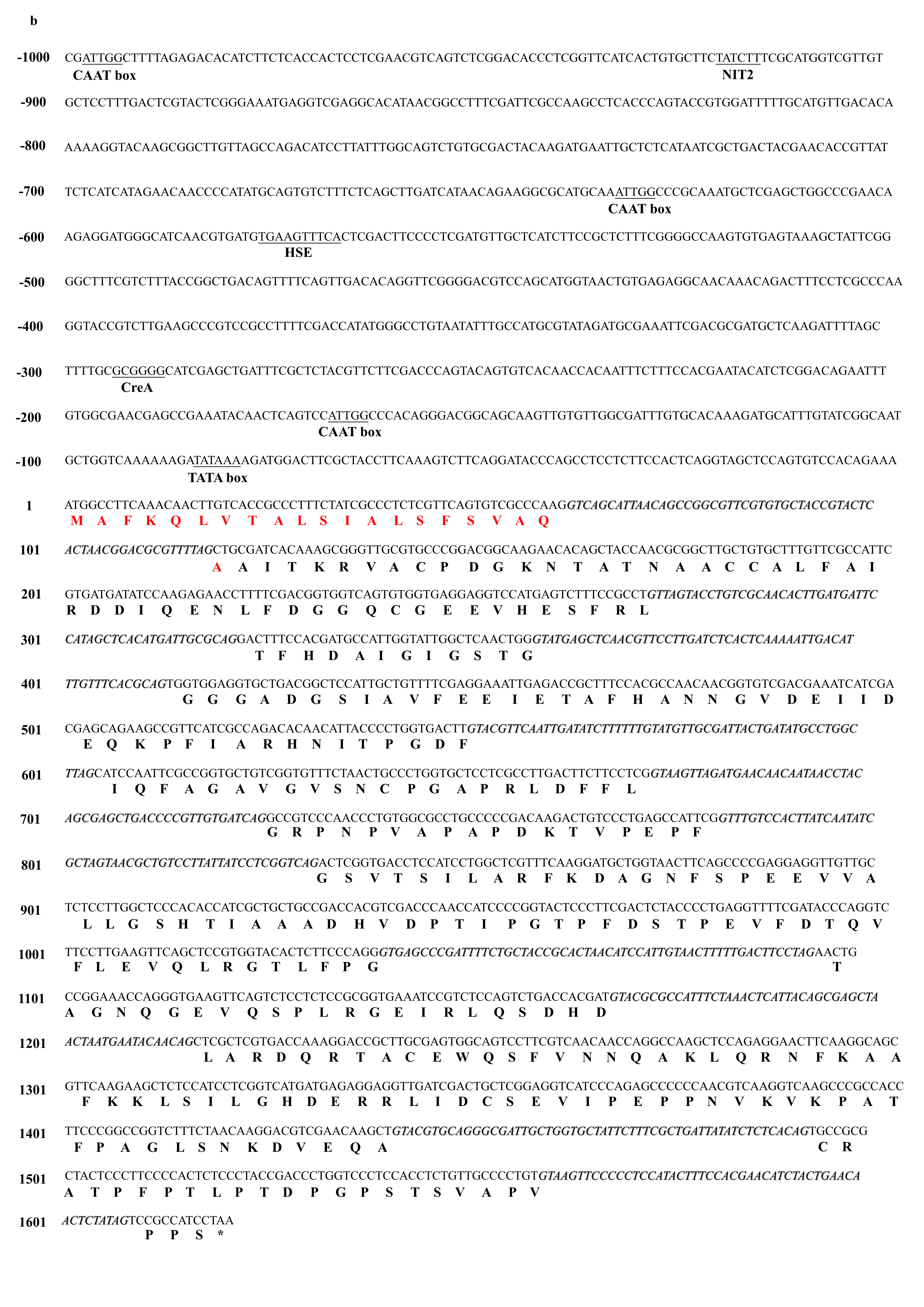


**Additional file 1：**The nucleotide and deduced amino acid sequences of the manganese peroxidase isoenzymes *Il*MnP1 (a) and *Il*MnP2 (b) of *I. lacteus* CD2. The signal peptide of the two manganese peroxidases was shown in red. The putative Cis-acting elements in the regulatory region are underlined. CreA: CreA-binding sites; XRE: xenobiotic-responsive elements; NIT2: NIT2 transcription factor consensus binding sequences; HSE: heat shock element.
