# Supplementary material for: Oxidation of a non-phenolic lignin model compound by two Irpex lacteus manganese peroxidases: evidence for implication of carboxylate and radicals
Source: Biotechnol Biofuels. 2017 Apr 21;10:103. doi: 10.1186/s13068-017-0787-z (PMC5399396; doi:10.1186/s13068-017-0787-z)
Supplement: Supplementary file 2 — Additional file 2. Purified recombinant IlMnP1 and IlMnP2 as analyzed by SDS-PAGE. Lanes: M, the protein molecular mass marker; 1, the purified IlMnP1; 2, the purified IlMnP2. [file 13068_2017_787_MOESM2_ESM.doc]

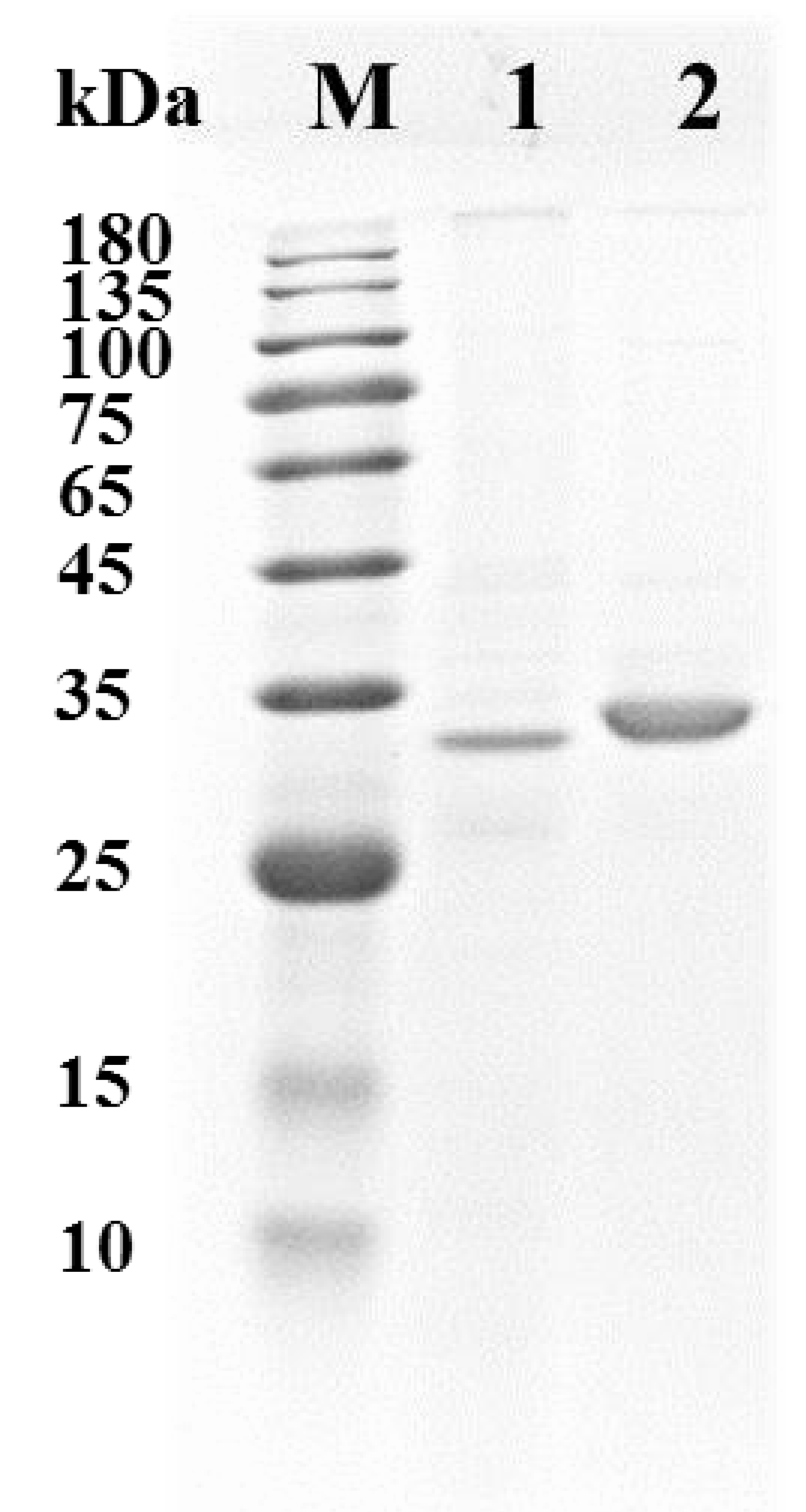


**Additional file 2:** Purified recombinant *Il*MnP1 and *Il*MnP2 as analyzed by SDS-PAGE. Lanes: M, the protein molecular mass marker; 1, the purified *Il*MnP1; 2, the purified *Il*MnP2.
