# Supplementary material for: Oxidation of a non-phenolic lignin model compound by two Irpex lacteus manganese peroxidases: evidence for implication of carboxylate and radicals
Source: Biotechnol Biofuels. 2017 Apr 21;10:103. doi: 10.1186/s13068-017-0787-z (PMC5399396; doi:10.1186/s13068-017-0787-z)
Supplement: Supplementary file 3 — Additional file 3. Effect of pH and temperature on the activity and stability of IlMnP1 and IlMnP2. (a) The pH-activity profiles. The activities at the pH optima were set as 100%. (b) The pH-stability profiles. The initial MnP activities before treatment were set as 100%. (c) The temperature-activity profiles. The activities at the temperature optima were set as 100%. (d) The temperature-activity profiles. The initial MnP activities before heat treatment were set as 100%. [file 13068_2017_787_MOESM3_ESM.doc]

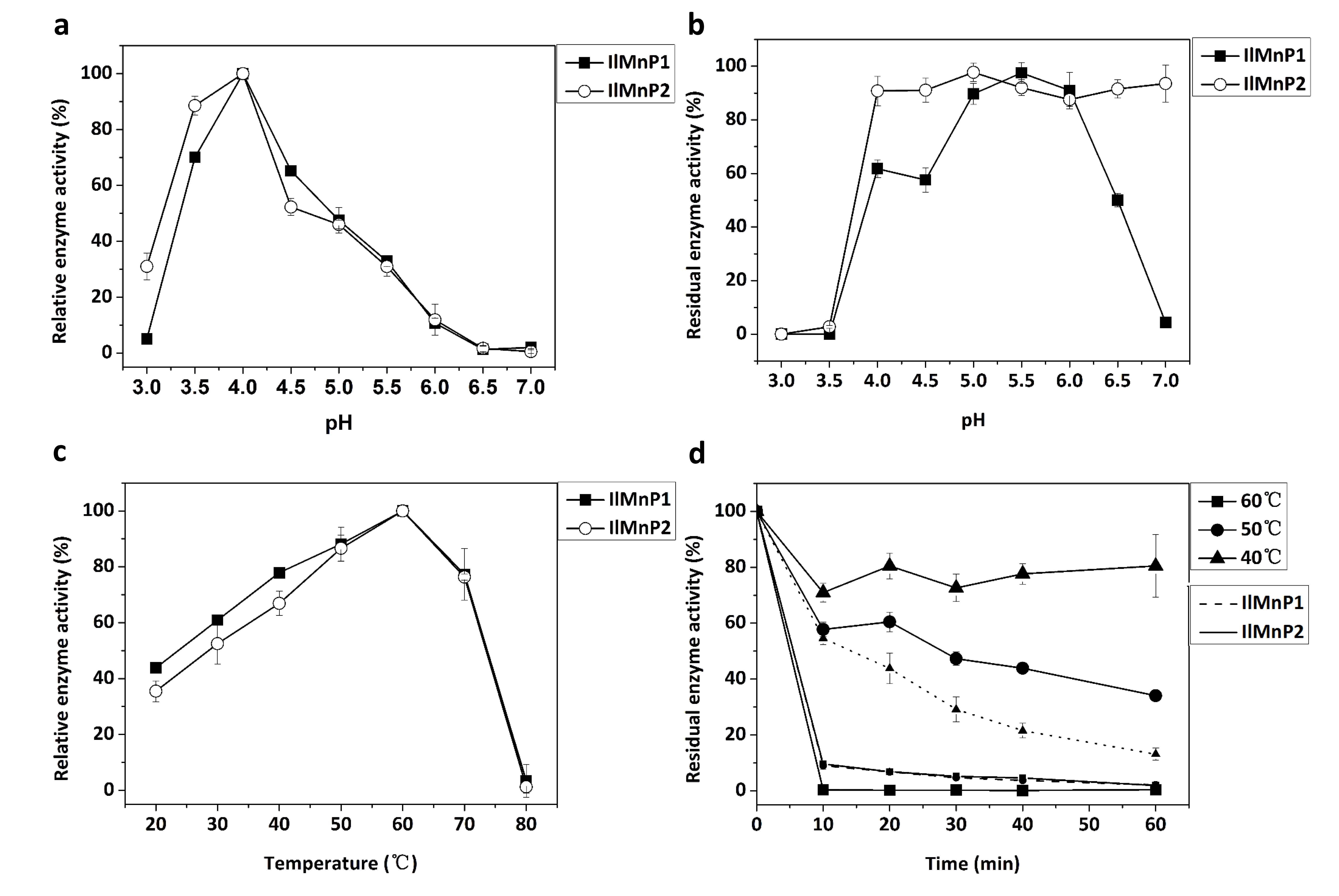


**Additional file 3:** Effect of pH and temperature on the activity and stability of *Il*MnP1 and *Il*MnP2. (a) The pH-activity profiles. The activities at the pH optima were set as 100%. (b) The pH-stability profiles. The initial MnP activities before treatment were set as 100%. (c) The temperature-activity profiles. The activities at the temperature optima were set as 100%. (d) The temperature-activity profiles. The initial MnP activities before heat treatment were set as 100%.
