# Supplementary material for: Oxidation of a non-phenolic lignin model compound by two Irpex lacteus manganese peroxidases: evidence for implication of carboxylate and radicals
Source: Biotechnol Biofuels. 2017 Apr 21;10:103. doi: 10.1186/s13068-017-0787-z (PMC5399396; doi:10.1186/s13068-017-0787-z)
Supplement: Supplementary file 4 — Additional file 4. Amino acid sequence alignment of IlMnP1 and IlMnP2 with selected MnPs, VPs, and LiPs. Inverted triangle: the cysteines that might form disulfide bridges; diamond: the structural Ca2+-binding residues; triangle: the active site histidine residues; hexagon: the acid residues forming the Mn2+ oxidation site; square: the tryptophan responsible for aromatic substrate oxidation. The GenBank accession numbers for these enzymes were: IlMnP1, KX620478; IlMnP2, KX620479; PoMnP2, KDQ32034.1; PoMnP4, 4BM1; PoMnP5, KDQ27903.1; PoMnP6, KDQ28248.1; PcMnP-H4, P19136.1; PeVP2, 2BOQ; PcLiP-H2, P11542.2; PcLiP-H8, AAB00798.1. [file 13068_2017_787_MOESM4_ESM.doc]

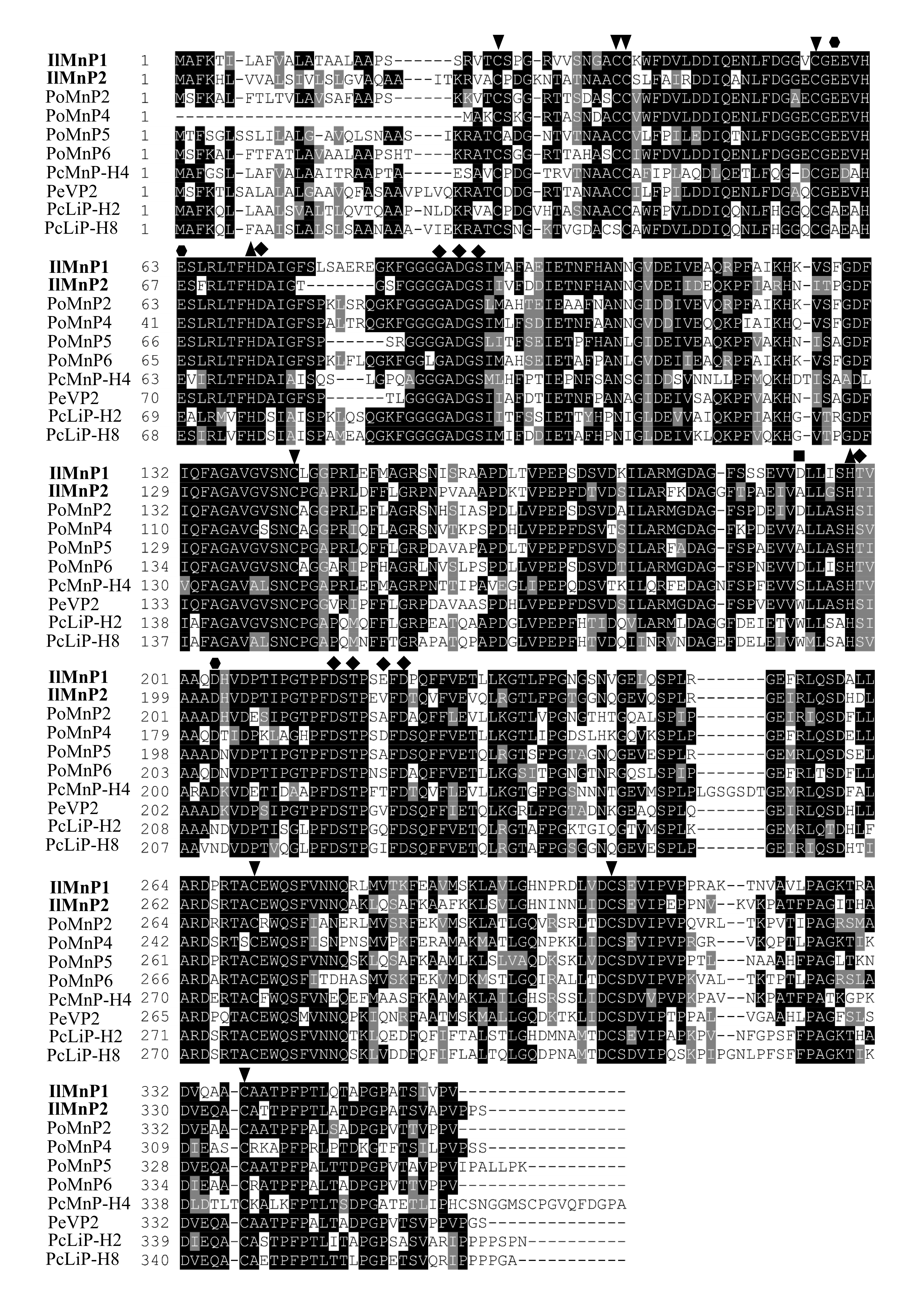


**Additional file 4：**Amino acid sequence alignment of *Il*MnP1 and *Il*MnP2 with selected MnPs, VPs, and LiPs. Inverted triangle: the cysteines that might form disulfide bridges; diamond: the structural Ca^2+^ -binding residues; triangle: the active site histidine residues; hexagon: the acid residues forming the Mn^2+^ oxidation site; square: the tryptophan responsible for aromatic substrate oxidation. The GenBank accession numbers for these enzymes were: *Il*MnP1, KX620478; *Il*MnP2, KX620479; *Po*MnP2, KDQ32034.1; *Po*MnP4, 4BM1; *Po*MnP5, KDQ27903.1; *Po*MnP6, KDQ28248.1; *Pc*MnP-H4, P19136.1; *Pe*VP2, 2BOQ; *Pc*LiP-H2, P11542.2; *Pc*LiP-H8, AAB00798.1.
