# Supplementary material for: Oxidation of a non-phenolic lignin model compound by two Irpex lacteus manganese peroxidases: evidence for implication of carboxylate and radicals
Source: Biotechnol Biofuels. 2017 Apr 21;10:103. doi: 10.1186/s13068-017-0787-z (PMC5399396; doi:10.1186/s13068-017-0787-z)
Supplement: Supplementary file 5 — Additional file 5. The non-phenolic lignin model compound veratryl alcohol was not oxidized by either IlMnP1 or IlMnP2 as analyzed by HPLC. The enzymes (0.5 U/mL IlMnP1 and IlMnP2, respectively) were incubated with VA in the acetate, citrate, lactate, or succinate buffer (50 mM, pH 5.0) with 1 mM Mn2+ at 30 °C for 48 h. [file 13068_2017_787_MOESM5_ESM.doc]

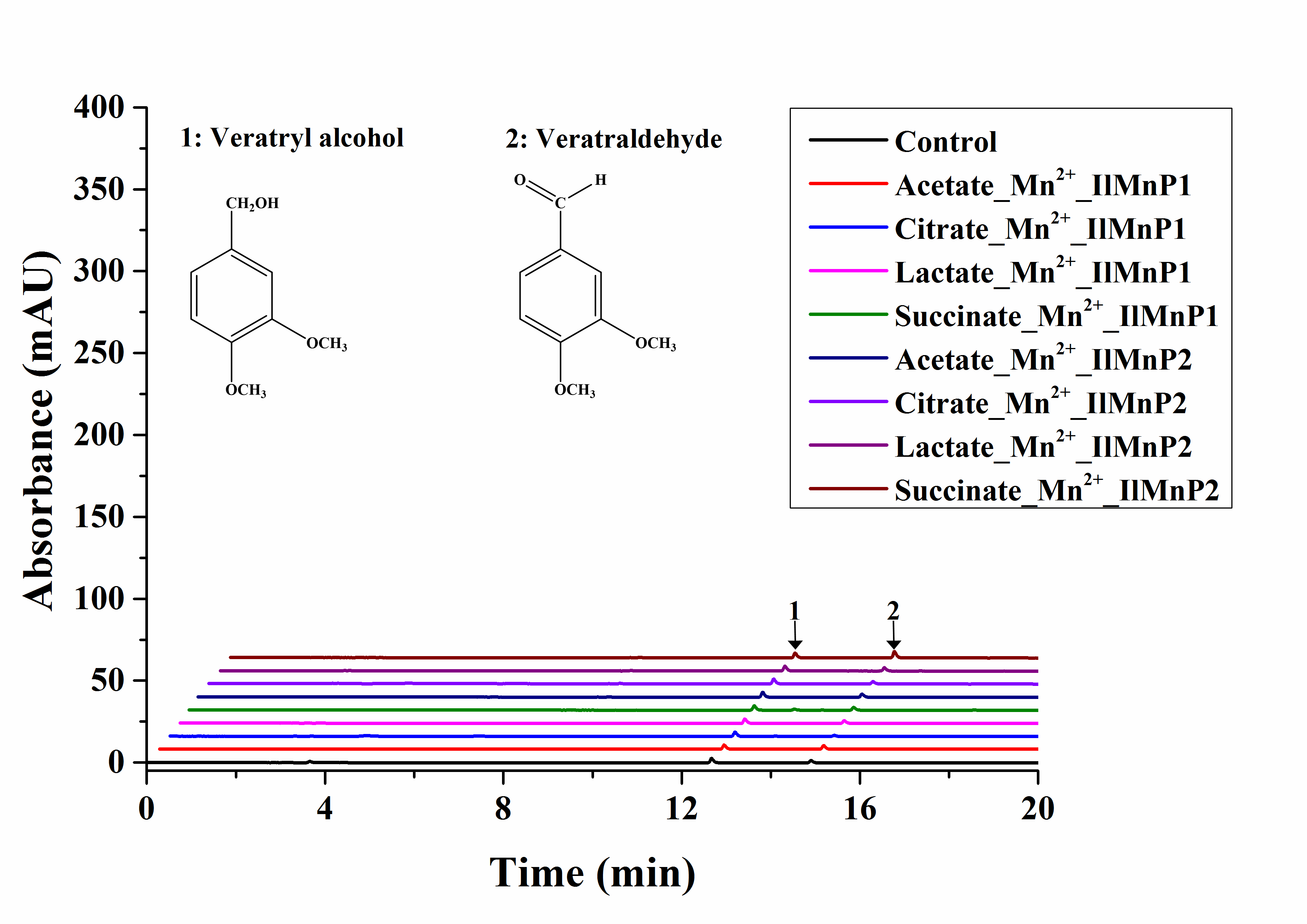


**Additional file 5：**The non-phenolic lignin model compound veratryl alcohol was not oxidized by either *Il*MnP1 or *Il*MnP2 as analyzed by HPLC. The enzymes (0.5 U/mL *Il*MnP1 and *Il*MnP2, respectively) were incubated with VA in the acetate, citrate, lactate, or succinate buffer (50 mM, pH 5.0) with 1 mM Mn^2+^ at 30 °C for 48 h.
