# Supplementary material for: Oxidation of a non-phenolic lignin model compound by two Irpex lacteus manganese peroxidases: evidence for implication of carboxylate and radicals
Source: Biotechnol Biofuels. 2017 Apr 21;10:103. doi: 10.1186/s13068-017-0787-z (PMC5399396; doi:10.1186/s13068-017-0787-z)
Supplement: Supplementary file 6 — Additional file 6. The product veratraldehyde steadily increased with a linear relationship to the concentrations of VA when oxidized by 0.5 U/mL IlMnP1 (a) or IlMnP2 (b). The reaction systems contained the malonate buffer (pH 5.0) and were incubated for 48 h in presence of 1 mM Mn2+. [file 13068_2017_787_MOESM6_ESM.doc]

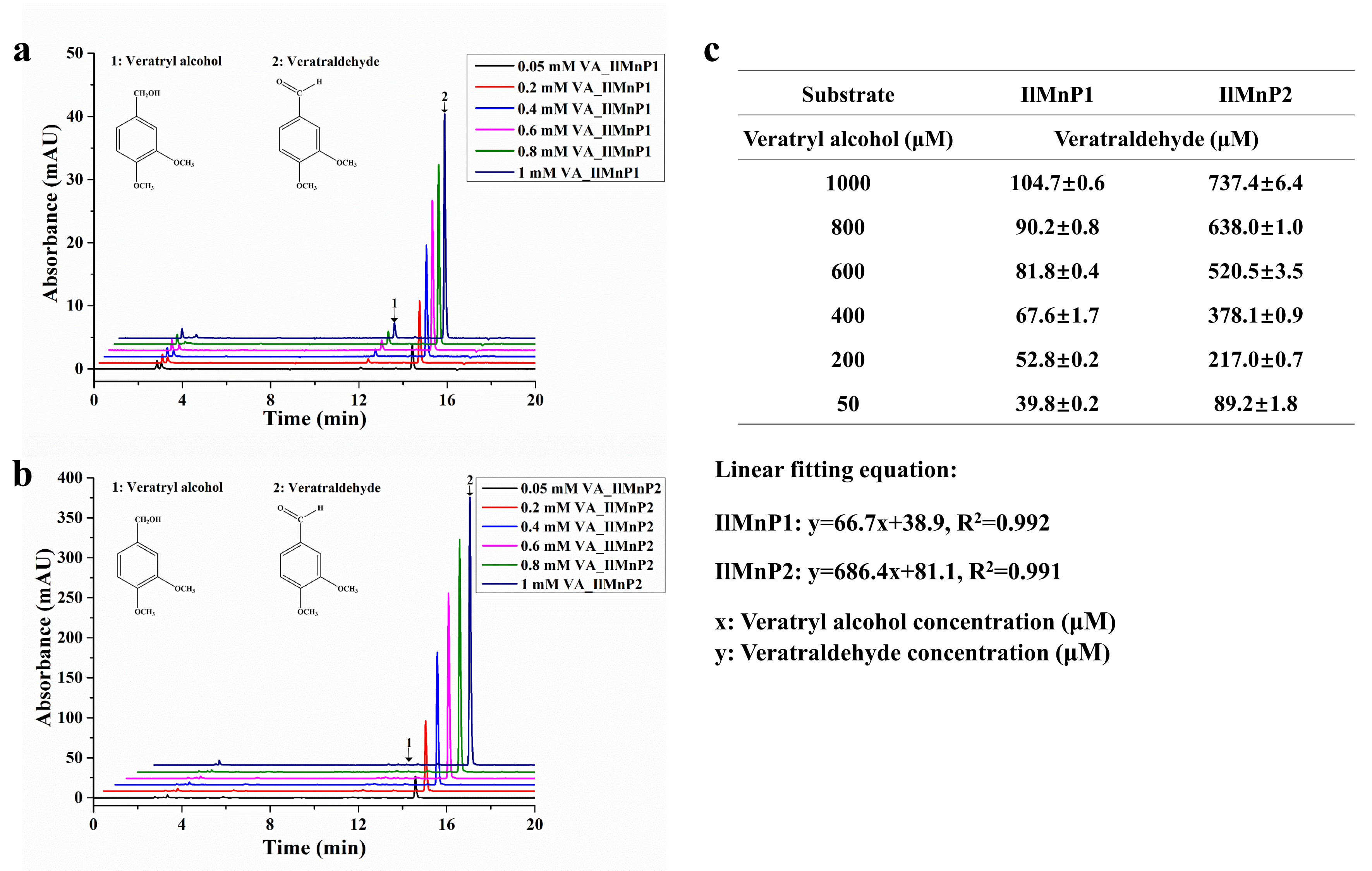


**Additional file 6：**The product veratraldehyde steadily increased with a linear relationship to the concentrations of VA when oxidized by 0.5 U/mL *Il*MnP1 (a) or *Il*MnP2 (b). The reaction systems contained the malonate buffer (pH 5.0) and were incubated for 48 h in presence of 1 mM Mn^2+^.
