# Supplementary material for: Oxidation of a non-phenolic lignin model compound by two Irpex lacteus manganese peroxidases: evidence for implication of carboxylate and radicals
Source: Biotechnol Biofuels. 2017 Apr 21;10:103. doi: 10.1186/s13068-017-0787-z (PMC5399396; doi:10.1186/s13068-017-0787-z)
Supplement: Supplementary file 7 — Additional file 7. Primers used in this study. [file 13068_2017_787_MOESM7_ESM.doc]

**Additional file 7：Primers used in this study.^a^**

| **Primer** | **Nucleotide sequence (5′→3′)** |
| --- | --- |
| MnP1-BamHI-F | CGC***GGATCC***GCACCCTCTTCTAGAGTGACATGCAGT |
| MnP1-NotI-R | TAAA***GCGGCCGC***TTACACAGGAACGATGGAGGTGGCG |
| MnP2-BamHI-F | CGC***GGATCC***GCAATCACCAAGCGTGTTGCTTGTCCT |
| MnP2-XhoI-R | CCG***CTCGAG***TTACGAGGGAGGGACAGGGGCGACAGA |

^a^The restriction sites are highlighted in bold and italic.
